# Beyond the screen: exploring digital health experiences of individuals affected by psoriasis – a qualitative interview study

**DOI:** 10.1186/s12889-025-24401-9

**Published:** 2025-09-09

**Authors:** Mert Ege Erbas, Stefanie Ziehfreund, Tilo Biedermann, Alexander Zink

**Affiliations:** 1 Department of Dermatology and Allergy, TUM School of Medicine and Health, Technical University of Munich, Biedersteiner Str. 29, 80802 Munich, Germany; 2 Division of Dermatology and Venerology, Department of Medicine Solna, Karolinska Institute, Stockholm, Sweden

**Keywords:** Digital health, Digital media, Physician-patient relationship, Psoriasis, Semi-structured interview

## Abstract

**Background:**

Psoriasis, a chronic inflammatory skin disorder, imposes a high burden on those affected, often leading to stigma and increased depression risk. With the increasing importance of digital media in medical contexts, there is a notable prevalence of misinformation and low-quality content. This study aims to explore the experiences of individuals affected by psoriasis regarding their disease-related digital media use.

**Methods:**

Semi-structured interviews with open-ended questions were conducted with psoriasis-affected people between August 2020 and January 2022 in Germany. The participants were recruited through digital media platforms, professional contacts, and in person at a university hospital department in southern Germany and were interviewed via video call. The recorded data was pseudonymized, transcribed verbatim, and analyzed using qualitative content analysis by Mayring which also allowed a quantitative evaluation of the category placements.

**Results:**

Eight participants (50% female) with a median age of 40.5 years (range: 25–80 years) were included. Four main categories emerged: (1) strengths and (2) difficulties of digital media in the context of psoriasis, (3) digital media in the context of the physician-patient relationship, and (4) suggestions for improvement. Commonly mentioned strengths were the positive impact on one’s well-being and the access to alternative therapy options. Frequently named problems were qualitative shortcomings and commercial interests. Most participants reported that digital media was not addressed in the physician-patient communication. Nevertheless, instances where it was discussed revealed predominantly negative reactions from physicians. Participants desired an increased availability of online resources and enhanced cooperation between digital media platforms and physicians.

**Conclusions:**

This study underscores the opportunities and challenges presented by digital media in managing psoriasis. Physicians should ensure that their patients access reliable platforms. Collaboration between physicians and affected individuals on digital media and adapting the traditional physician-patient relationship to an increasingly digitalized world are suggested to enhance patient care.

**Supplementary Information:**

The online version contains supplementary material available at 10.1186/s12889-025-24401-9.

## Background

Psoriasis is a common inflammatory skin disease with a prevalence between 0.5% and 11.4% in adults worldwide [1] and is caused by several genetic and environmental risk factors and immunological pathologies [2]. Possible triggers can be stress, an infection, smoking, alcohol consumption, and medication, e.g., lithium. Depending on the psoriasis severity, presence of psoriasis arthritis, comorbidities, and patient’s preferences, treatment options are topical therapy, phototherapy, oral-systemic therapy (e.g., methotrexate), and biologics, which are all considered effective [3]. Compared to the general population, individuals affected by psoriasis experience increased mental burden due to stigma [4], making them more susceptible to depression and impaired happiness [5].

Individuals with dermatological conditions increasingly use digital media for health issues [6–9], especially young and highly educated people affected by skin conditions with a high social burden [8]. Digital media refers to the dissemination of information in the form of digital data, encompassing various content types — including audio, video, graphics, and text — delivered through digital devices such as computers, tablets, and smartphones. Examples include social media platforms (e.g., Facebook), video streaming services (e.g., YouTube), digital radio stations, and online encyclopedic resources (e.g., Wikipedia) [10]. Moreover, individuals affected by psoriasis have shown a higher risk for general internet addiction, particularly individuals of younger age, not being in medical care, and not being in a relationship [11]. Thus, adequate e-health literacy is necessary [12]. The concept of e-health literacy is defined as an individual’s ability to search for, access, comprehend, critically assess, and apply health-related information from digital sources in order to make informed decisions or take appropriate action concerning health issues [12]. However, both younger [13] and older [14] individuals show low e-health literacy, emphasizing the need to improve their e-health literacy to ensure a safe patient journey. The patient journey represents the chronological sequence of phases starting with the first symptoms [15] which is positively influenced by the individuals’ involvement in the medical process [16].

Multiple studies evaluated the information quality of digital media regarding psoriasis. Roche et al. assessed the online information and conspiracy theories via Google and PubMed and considered the misinformation related to psoriasis on social media and other websites as pervasive. Key aspects of false information included victim-blaming, contagion, and denigration of conventional therapy [17]. The quality of psoriasis information on social media platforms like Facebook [18], YouTube [19], or Instagram [20] have been found to be deficient across all mentioned platforms, with even dangerous content [19] that exists on digital media. A study evaluating the dangers of social media for patients with atopic dermatitis even conveyed that a patient discontinued the prescribed treatment based on social media influence [21]. However, there are also studies suggesting benefits from digital media regarding health-related issues, for example, the promotion of cancer prevention and education [22].

A quantitative study suggested that digital media could be used by physicians as an opportunity to recommend high-quality platforms to patients, thereby enhancing patient care [23]. In addition, it was discussed that psoriasis patients in clinical settings generally place greater trust in the expertise of their physician when there exists a discrepancy between information found on social media and the advice provided by a physician. However, it is common for patients to refrain from sharing the findings of their online research with their physicians [24].

All of these studies included individuals from the clinical setting [23, 24]. Additionally, as no qualitative study has examined the experiences of psoriasis-affected people on digital media, a qualitative approach to this issue with individuals inside and outside of clinical settings was chosen in this study.

This study aims to explore the experience of disease-related digital media use in the context of psoriasis and how digital media affects individuals’ care from the perspective of psoriasis-affected individuals inside and outside clinical settings.

## Materials and methods

### Study design

The established standards for reporting qualitative research (i.e., COREQ [Consolidated Criteria for Reporting Qualitative Research] checklist) were followed in this study (see Additional file 1) [25]. Semi-structured interviews with individuals affected by psoriasis in Germany were carried out between August 2020 and January 2022. Participants were informed about the nature of the study, the pseudonymization of the data, their rights as participants in layman’s terms, and any questions regarding the study were answered via email or video call before written informed consent were given by the participants.

### Study population

Eligible for inclusion were people aged ≥ 18 years with self-reported psoriasis and the ability to communicate effectively in German. There was no criterion for exclusion. The participants were recruited through convenience and purposive sampling via social media (e.g., via Facebook) and on German online psoriasis self-help platforms (i.e., “Psoriasis-Netz” and “Farbenhaut”), in-person at the Department of Dermatology and Allergy at the Technical University of Munich, southern Germany, and through professional contacts. Most of the individuals were contacted either via email or direct message on social media by one male researcher (ME, medical student and doctoral candidate, trained in qualitative research). Two participants reached out to ME after reading a recruitment post on online platforms. Subsequently, the appointments for the interviews were scheduled by the interviewer (ME) and the participants. The participants were not financially compensated. The interviewer did not know any of the participants prior to the study and no participant dropped out. Beforehand the interviewer informed every participant about his characteristics (name, current position) and the research goals.

### Instrument and measures

A semi-structured interview guide (see Additional file 2) with open-ended questions was developed by an interdisciplinary team including a dermatologist, two epidemiologists, and two medical students and was based on the recent literature [26–31] and the manual for conducting interviews published by Helfferich [32]. The guide consisted of main questions and probing questions and was subdivided into four parts. The first part covered the personal use of digital media. In the second part, the participants were asked about their perception of the online behavior of other people affected by psoriasis. The last two parts contained questions about the difficulties and suggestions for improvement regarding digital media. The interview guide was pilot-tested and reviewed by one patient resulting in minor changes regarding the wording of some questions for better comprehensibility. All interviews were carried out one-on-one online via video call by one researcher (ME) and were audio-recorded. All interviewed people were at home and no one else was present during the interviews. After every interview, the interviewer took field notes about the atmosphere and disturbances throughout the interview with none having occurred. No repeat interviews were carried out.

### Data analysis

The audio-recorded interviews were transcribed verbatim and manually by the interviewer. The interviewees did not receive the written transcripts. Participants’ names were pseudonymized by replacing the name with P and a number which represents the chronological order of the interviews. The transcripts were analyzed using qualitative content analysis by Mayring [33] with the qualitative data analysis software MAXQDA 2022 (VERBI Software, Berlin, Germany). First, deductive categories were determined based on the relevant topics of the interview guide. Then, two researchers (ME and SZ) initially read through the same three randomly picked interviews to classify further inductively emerged categories based on the transcripts. Both researchers compared their findings and discussed the main themes.

The two researchers used the category system during independent coding of the same three transcripts that were selected before. Hereafter, an interrater reliability was calculated in MAXQDA, based on Cohen’s Kappa resulting in a high level of agreement (*k* = 0.91). The remaining 5 interviews were coded individually by ME with two codes (“simpler way of participating in events” and “cooperation with physicians”) being added to the codebook, since these aspects have not been discussed in the three randomly picked interviews mentioned before and were considered important by the interviewer. Data saturation was determined to have been achieved once the interviewers’ subjective judgment indicated that no further obtainable new information was available. In addition, a quantitative evaluation of the category placements was undertaken.

## Results

Eight individuals (4 male and 4 female) with a median age of 40.5 years (range: 25–80 years) participated in this study. The participants’ engagement in online research varied from daily to no disease-related digital media use (Table 1).

**Table 1:** The characteristics of the participants (*n* = 8)

ID	Age (years)	Gender	Internet research behavior
P1	27	Female	Uses digital media for medical reasons daily. She has an Instagram account about her journey with psoriasis and she exchanges information with other affected people online. She informs herself about nutrition and alternative care. She apparently knows about the recent studies about psoriasis.
P2	59	Female	Uses especially Facebook daily. She does online research about her disease and alternative care. In addition, she exchanges information with other affected individuals. She does not read recent studies.
P3	49	Male	No disease-related digital media use.
P4	63	Male	Regularly uses social media and ‘Psoriasis-Netz’ (German psoriasis self-care platform) for disease-related issues.
P5	28	Male	Uses the digital media daily for his disease. For that, he uses social media and reads online journals and studies about psoriasis.
P6	80	Female	Informs herself on digital media about her disease 4–5 times a month.
P7	25	Female	Does disease-related research on digital media every 2–3 months or in case of symptoms.
P8	32	Male	Hardly uses digital media because of his disease because he has no symptoms due to his medication. He does not want to deal with his disease if he does not have to. If at all, he looks up a physician’s phone number now and then. In the past, online research was more important to him.

The interviews lasted between 12 and 48 min (median: 28.5 min). The following four categories emerged as the key aspects: (1) strengths of digital media in the context of psoriasis, (2) difficulties of digital media in the context of psoriasis, (3) digital media in the context of the physician-patient relationship, and (4) suggestions for improvement (Table 2).

**Table 2:** The main categories and codes with the quantitative analysis for each category and code

Category (number of codes)	Codes within the category (number of codes)
1. Strengths of digital media in the context of psoriasis (25)	- Positive impact on the peoples’ well-being: ➔ Reassurance by other individuals (8) ➔ Reassurance by the knowledge gained through digital media (2) ➔ Relativization of psoriasis by research (1) - No stigmatization online (6) - Strengthening of the more mature handling of the disease (1) - Access to other therapy options (6) - Simpler way of participating in events (1)
2. Difficulties of digital media in the context of psoriasis (67)	- Negative influence on the well-being (9) - Information overflow (9) - Lack of quality (18) - Hidden commercial interests (13) - Frustration over therapy promises (4) - Unintelligible terms (6) - Old fashioned platforms (4) - Hiding of psoriasis online (4)
3. Digital media in the context of the physician-patient relationship (36)	- Education by the physician (10) - Negative view of the physicians regarding the digital media (9) - More trust in digital media (2) - No online research because of trust in physicians (11) - Preparation to improve the physicians’ appointment (2) - Importance of physicians’ appointment (2)
4. Suggestions for improvement (32)	- More structured information (6) - Expand of the digital media offer (11) - Cooperation with physicians (6) - Illustration of medical care (3) - Neutral controlling instance (6)

### Strengths of digital media in the context of psoriasis

Aspects of digital media contributing positively to their well-being were mentioned, e.g., a feeling of reassurance after reading about other individuals’ histories, and not feeling alone with the disease:

*“You see for example: ‚Ok*,* she literally has the same skin just as I do.‘ So*,* I have never seen that before*,* because you never see that anywhere in your spare time.“ – P1*.

Also, it was specified that gaining knowledge after researching psoriasis led to a positive feeling. In addition, one participant mentioned that watching videos of people or influencers who had a worse blow of fate made him put his disease into perspective. He described that this has strengthened and inspired him mentally.

Almost all participants highlighted the lack of stigmatization of people with psoriasis online as a positive aspect of digital media platforms, whereas in real-life they often experienced prejudice and stereotypes. Moreover, it was noticed that research on digital media empowered the participants to deal with psoriasis more responsibly and to understand medical contexts:

*“Of course*,* it helps me to be more mature when dealing with the disease. For example*,* in conversations with dermatologists I can assess things more and I don’t trust them blindly.” – P5*.

Some participants described that using digital media provides access to other therapy options and makes it easier for them to participate in events e.g., self-help meetings or online seminars. One participant affected severely by psoriasis arthritis and thus immobility appreciated the online events offered especially during the COVID-19 pandemic.

### Difficulties of digital media in the context of psoriasis

Participants who have mentioned a positive influence on their well-being before also raised concerns about the potential negative impact of digital media on their well-being. Negative reports about possible side effects or the lack of success in therapy from other affected individuals caused overthinking and mental stress. Consequently, some of the participants reported that they stopped researching digital media. Moreover, the information overload and the poor quality of platforms, including outdated data and dangerous misinformation on social media were expressed as issues. Additionally, the potential influence of commercial interests was subject to critical examination by the participants:

*“For example*,* stuff on the market that promises to heal psoriasis. They hornswoggle you and the stuff costs a lot of money and has no effect. So that is a very big threat.” – P2*.

The numerous promises about the efficacy of various medications coupled with repeated disappointments regarding their efficacy greatly complained the participants:

*“Especially when you read empty promises and you think: ‘This could help me.’ Then you are excited and you are at the end of one’s rope. But it does not help at all. It is very disappointing every single time.” – P7*.

It was also mentioned that medical terminology is problematic because lots of individuals do not know words like ‘indication’ or ‘anamnesis’.

The difficulty of revealing their psoriasis lesions on social media was discussed and participants admitted to intentionally sharing only photos online with no visible psoriasis lesions. One of them emphasized how people generally hide allegedly unattractive aspects, contributing to an idealized online environment. Overcoming this pressure and embracing their authentic selves proved challenging.

### Digital media in the context of the physician-patient relationship

Nearly all participants claimed that no physician has ever informed and educated them about any disease-related digital media. Beyond that, it was mentioned that some physicians even have concerns about digital media in the context of psoriasis:

*“I think the physician is too wary that I receive false information.” – P2*.

It was also discussed that the participants felt judged by physicians and preferred not to mention their research on digital media:

*“There is a block within the physicians. Like they say: ‘Oh, Dr. Google.’ Then I do not dare to say or ask anything. ‘Oh well, she has read something. She thinks she is smart now.’ Then it is not fun to say anything. I rather shut my mouth.” – P6.*

In addition, one participant felt like her physician did not provide enough information about all therapy options, especially about alternative care. So, she consulted digital media instead.

Furthermore, one participant explained that researching digital media is used to prepare for a medical appointment to make the most efficient use of limited time with a medical specialist. He added that as a well-informed patient, he could have an opinion for example about proposed therapy plans right away, instead of waiting for months until the next appointment. The same person recounted that consulting a physician was still very important:

*“I think the internet is extremely powerful. It does not replace a physician’s appointment. Even if I had researched for 5 years*,* I would not have the knowledge of a dermatologist. Technology can never replace a medical specialist because it is about expertise and experience” – P5*.

However, participants also described not researching digital media because they trusted their physicians or the success of their therapy.

### Suggestions for improvement

Based on the disadvantages that were described, the participants suggested improvements for digital media platforms, namely more structured information. It was mentioned that a summary or overview of psoriasis itself with all therapy options and the latest scientific findings would be helpful. In addition, the participants proposed an expansion of the digital media offer for psoriasis-affected people, e.g., creating more chatrooms, which would lead to a better exchange between the affected people.

A participant added that having a neutral controlling instance and a neutral moderator would improve the digital media regarding psoriasis-affected people. One participant who does not use digital media for health reasons highlighted that physicians should be more involved in psoriasis-related digital media platforms:

*“It would be great if physicians were behind online platforms. Then physicians could evaluate: ‘Look*,* try this medication or this.’ And you do not have to sit in the physician’s practice for hours hoping that you can try another medication.” – P3*.

Furthermore, it was reported that medical care options should be more illustrated so that affected individuals know which physician’s practice has any capacity.

## Discussion

### Principal results and comparison with prior work

This study provides in-depth insight into psoriasis-related digital media use, as perceived by individuals affected by psoriasis to strengthen the potential of use and counteract disadvantages in targeted interventions. The present study revealed perceived advantages of digital media like a positive impact on well-being, the absence of stigmatization, and access to other therapy options. However, disadvantages predominated, including e.g., the poor quality of the online platforms or the hidden commercial interests of online services. Participants highlighted a deficiency in physician education concerning digital media within the physician-patient relationship. To improve digital media in the context of psoriasis, the participants suggested expanding the online offer regarding psoriasis and including physicians in the development of digital media content.

A review indicates that psoriasis-affected individuals experience stigmatization in real-life settings [34]. However, the absence of such stigma in digital media observed in this study may be attributed to photo editing practices on social media. Research by Ozimek et al. suggests that photo editing is negatively linked to self-esteem [35]. In addition, participants mentioned that they only post photos on social media with no visible psoriasis lesions which does not offer the possibility of being stigmatized. Moreover, some participants of this study use psoriasis self-help platforms or social media, e.g., Facebook where lots of private psoriasis groups can be found. Thus, the people affected by psoriasis are among themselves and do not experience external stigmatization. However, it was previously described that addressing psoriasis on digital media had the potential to reduce stigmatization [36].

The qualitative shortcomings discussed in the present study are in accordance with other studies where up to two-thirds of psoriasis-related videos on YouTube and information on Facebook were considered misleading or even dangerous [18, 19]. In the context of the growing importance of digital media for psoriasis during the last years [9] and the large burden of disease among psoriasis-affected individuals [5], digital media should be a safe space and an opportunity to improve the mental health of those affected by having access to valid information and a place to exchange experiences with other affected individuals. To ensure the reliability of digital media content for psoriasis-affected people, impartial regulatory bodies including medical experts could be established to review such content. Additionally, high-quality digital media platforms could be prioritized in search engine rankings, increasing their visibility and likelihood of use.

While the participants’ online behavior in the present study may be considered unremarkable, physicians and creators of digital media platforms should be cognizant of the heightened prevalence of internet addiction among psoriasis patients [11]. Given that this type of addiction can lead to poor sleep quality and fatigue [37] subsequently leading to stress—a potential trigger for psoriasis— [3], efforts should be made to break this detrimental cycle. This is particularly relevant as individuals may turn to digital media to cope with disease-related burdens, potentially increasing the risk of problematic usage patterns.

While prior studies implicated an improvement in the physician-patient relationship through disease-related digital media use [38, 39], the present study implicated rather the opposite. These opposing findings could be explained by the different samples as individuals with potentially lethal diseases were recruited who might have an increased trust towards their physician [38]. In addition, one of these studies also included individuals without chronic diseases [39]. A potential reason for the negative impact in our work might be that some physicians may be used to a more patriarchal model of the physician-patient relationship and feel that their competence may be questioned by their patients’ research. To meet the evolving needs of informed patients, the traditional physician-patient relationship needs restructuring. A medical approach that prioritizes the physician-patient relationship and incorporates the patient’s perspective is crucial [40]. A deliberative and participatory model, contrasting the physician-centered and paternalistic models, is recommended for patients who gather online information [41]. In this model, physicians educate and support patients, engaging them in a dialogue to understand and select from available options during the decision-making process [42].

The concerns of physicians regarding digital media reported by patients in our study are in line with two studies proving the negative attitude and lack of understanding among physicians when it comes to patients bringing online information to a consultation [43, 44]. Recommending specific digital platforms or physician involvement could ensure access to high-quality, evidence-based information, enhancing patient care. Consequently, patients may engage more responsibly with their conditions, fostering better comprehension and enabling shared decision-making. Moreover, using digital media may alleviate time pressure during appointments, as individuals using digital media tend to consult physicians less for minor issues [45]. Additionally, providing information about accessible dermatological care centers could address the challenge of limited access highlighted in various studies [46–50]. This approach may also facilitate the integration of untreated individuals into the healthcare system, thereby improving their path to proper medical care.

In addition, there are also studies underlining that in-person consultation and the use of digital media for health care should be combined [6]. In an editorial by an American dermatologist, many advantages for dermatologists using social media were listed [51] showing that not only patients profit from more digital health care, but physicians as well. One study indicated that a small number of dermatology residents received social media training. However, the majority expressed a desire for such training, as they perceive social media platforms as crucial tools, particularly for engaging with and educating patients [52]. These desires could be addressed by elaborating strategies to integrate social media training in the residents’ education. As dermatological organizations and journals increasingly use social media [53], it is essential to ensure that patients have sufficient e-health literacy [12] to improve specialized health care and the physician-patient relationship. This could be achieved by providing easily accessible information on how to find high-quality, evidence-based information on digital media and how to identify qualitative shortcomings. For this approach, health insurers and politicians could work collaboratively to support the patients’ education and e-health literacy.

### Limitation and strengths

Recall bias is likely to be present when people talk about past experiences, and participants may be more likely to give socially desirable responses [54]. Online interviews include challenges like the interviewer’s and participants’ technical skills and stable Internet connection. Participants’ answers in online interviews are usually shorter and lack contextual information [55]. However, in-person interviews are assumed to be only slightly superior to video calls [56]. The qualitative nature of the study implies that the results cannot be generalized. Another limitation is the limited sociodemographic information collected about the participants. Aside from age and gender, detailed data on socioeconomic status or educational background were not systematically gathered, which may constrain the contextual interpretation of the findings. Furthermore, the study was geographically limited to Germany, which may affect the generalizability of findings to other cultural or healthcare system contexts. Some interviews were conducted during the COVID-19 pandemic, which may have intensified participants’ reliance on digital media for health-related information and support. Moreover, the COVID-19 pandemic likely increased public awareness about misinformation and may have influenced participants’ vigilance and critical assessment of health information in digital media. Although this contextual factor was not specifically investigated in our study, it should be considered when interpreting participants’ digital media behaviors during the study period. Even though the sample is small, heterogeneity was achieved for age, gender, and internet search behavior due to the purposive sampling procedure resulting in a very diverse collection of opinions. Additionally, that heterogeneity was further augmented by not only recruiting individuals inside clinical settings, but also from digital media platforms, thereby encompassing individuals who have limited contact with healthcare professionals or infrequent physician appointments. Moreover, content saturation was reached [57]. When researching the use of digital media or the Internet for health-related information, surveys often capture quantitative data [6, 39, 58] but may miss subjective viewpoints. In our study, we opted for semi-structured interviews to capture these subjective aspects. To integrate both qualitative and quantitative analysis, we then used qualitative content analysis by Mayring as a mixed-methods approach, which enhanced the analytical strength of our findings [59].

## Conclusions

The study emphasizes the potential benefits of digital media for individuals with psoriasis, highlighting the need to address challenges and make improvements for a better online experience. Physicians should ensure patients access evidence-based information and legitimate digital media. Collaborative efforts between patients and physicians on digital media platforms and adapting the traditional physician-patient relationship to an increasingly digitalized world are recommended to enhance patient care. This study also highlights the importance of enhancing patients’ e-health literacy and addressing physicians’ desire for social media training. In addition, future studies should elaborate strategies to ensure the quality of health-related content on digital media and to improve the accessibility of evidence-based and reliable resources in digital media. These findings could be used to enhance or, if necessary, to expand the existing digital media offerings. Moreover, future research should explore how e-health literacy interventions can be tailored to individuals with chronic skin conditions, considering their emotional needs and patterns of engagement in digital media. Comparative studies across different countries or health systems could help identify structural factors that shape health-related digital media use. In addition, further investigation is needed into the risks and benefits of peer support in digital media, especially in relation to problematic digital media use and the spread of misinformation.

## Supplementary Information

Additional file 1

Additional file 2

## Data Availability

The datasets used and/or analyzed during the current study are available from the corresponding author on reasonable request.

## Abbreviations

COREQ: Consolidated Criteria for Reporting Qualitative Research
