# Supplementary material for: Beyond the screen: exploring digital health experiences of individuals affected by psoriasis – a qualitative interview study
Source: BMC Public Health. 2025 Sep 9;25:3041. doi: 10.1186/s12889-025-24401-9 (PMC12418609; doi:10.1186/s12889-025-24401-9)
Supplement: Supplementary file 1 — Additional file 1 [file 12889_2025_24401_MOESM1_ESM.docx]

**Additional file 1** *COREQ-Checklist based on Tong A, Sainsbury P, Craig J. Consolidated criteria for reporting qualitative research (COREQ): a 32-item checklist for interviews and focus groups. Int J Qual Health Care. 2007 Dec;19(6):349-57. doi: 10.1093/intqhc/mzm042. Epub 2007 Sep 14. PMID: 17872937.*

| No | Item | Guide question/description | Line |
| --- | --- | --- | --- |
| **Domain 1: Research team and reflexivity** | | | |
| Personal Characteristics | | | |
| 1 | Interviewer/facilitator | Which author/s conducted the interview or focus group? | 133 |
| 2 | Credentials | What were the researcher’s credentials? E.g. PhD, MD | 118 |
| 3 | Occupation | What was their occupation at the time of the study? | 118 |
| 4 | Gender | Was the researcher male or female? | 118 |
| 5 | Experience and training | What experience or training did the researcher have? | 118-119 |
| Relationship with participants | | | |
| 6 | Relationship established | Was a relationship established prior to study commencement? | 121-112 |
| 7 | Participant knowledge of the interviewer | What did the participants know about the researcher? e.g. personal goals, reasons for doing the research | 122-123 |
| 8 | Interviewer characteristics | What characteristics were reported about the interviewer/facilitator? e.g. Bias, assumptions, reasons and interests in the research topic | 122-123 |
| **Domain 2: Study design** | | | |
| Theoretical framework | | | |
| 9 | Methodological orientation and  Theory | What methodological orientation was stated to underpin the study? e.g. grounded theory,  discourse analysis, ethnography, phenomenology, content analysis | 127-128, 142-143 |
| Participant selection | | | |
| 10 | Sampling | How were participants selected? e.g. purposive, convenience, consecutive, snowball | 113-120 |
| 11 | Method of approach | How were participants approached? e.g. face-to-face, telephone, mail, email | 117-120 |
| 12 | Sample size | How many participants were in the study? | 158 |
| 13 | Non-participation | How many people refused to participate or dropped out? Reasons? | 122 |
| Setting | | | |
| 14 | Setting of data collection | Where was the data collected? e.g. home, clinic, workplace | 133-135 |
| 15 | Presence of non-participants | Was anyone else present besides the participants and researchers? | 135 |
| 16 | Description of sample | What are the important characteristics of the sample? e.g. demographic data, date | 158-160 |
| Data collection | | | |
| 17 | Interview guide | Were questions, prompts, guides provided by the authors? Was it pilot tested? | 132-133 |
| 18 | Repeat interviews | Were repeat interviews carried out? If yes, how many? | 137 |
| 19 | Audio/visual recording | Did the research use audio or visual recording to collect the data? | 133-134 |
| 20 | Field notes | Were field notes made during and/or after the interview or focus group? | 135-137 |
| 21 | Duration | What was the duration of the interviews or focus group? | 162 |
| 22 | Data saturation | Was data saturation discussed? | 154-155 |
| 23 | Transcripts returned | Were transcripts returned to participants for comment and/or correction? | 139-140 |
| **Domain 3: Analysis and findings** | | | |
| Data analysis | | | |
| 24 | Number of data coders | How many data coders coded the data? | 144-147 |
| 25 | Description of the coding tree | Did authors provide a description of the coding tree? | 166 |
| 26 | Derivation of themes | Were themes identified in advance or derived from the data? | 143-146 |
| 27 | Software | What software, if applicable, was used to manage the data? | 142-143 |
| 28 | Participant checking | Did participants provide feedback on the findings? | 139-140 |
| Reporting | | | |
| 29 | Quotations presented | Were participant quotations presented to illustrate the themes / findings? Was each  quotation identified? e.g. participant number | 171-172, 181-183, 197-198, 201-203, 215, 218-220, 228-231, 245-247 |
| 30 | Data and findings consistent | Was there consistency between the data presented and the findings? | 168-249 |
| 31 | Clarity of major themes | Were major themes clearly presented in the findings? | 166 |
| 32 | Clarity of minor themes | Is there a description of diverse cases or discussion of minor themes? | 166 |
