# Supplementary material for: Beyond the screen: exploring digital health experiences of individuals affected by psoriasis – a qualitative interview study
Source: BMC Public Health. 2025 Sep 9;25:3041. doi: 10.1186/s12889-025-24401-9 (PMC12418609; doi:10.1186/s12889-025-24401-9)
Supplement: Supplementary file 2 — Additional file 2 [file 12889_2025_24401_MOESM2_ESM.docx]

**Additional file 2** *Topic guide for semi-structured interviews (shortened for publication)*

| **Main question** | **Probing question** | **Aim** |
| --- | --- | --- |
| How would you describe your online behavior regarding psoriasis? | - Which internet sources do you use regularly? (e.g., Wikipedia, forums, social media, health websites) - What aspects of psoriasis do you research? - Does online information help you dealing with the disease? - Which topics do you research online rather than discuss with your physician? - How does your online behavior help you with the mental burden of the disease? - What are the main reasons of your online media usage regarding psoriasis? - How important is connecting with other affected people online to you? - How does your online research behavior influence your physician-patient relationship? | Self-reflection of their own online research behavior |
| How do you perceive the online research behavior of other psoriasis patients? | - How would you describe the networking of the psoriasis community? - To what extent do you network with other affected people? - Which online platforms are most frequently used by psoriasis-affected individuals and why? | Understanding the individuals’ perception of other psoriasis-affected people online |
| Which difficulties do psoriasis patients have regarding online media? | - To what extent do you think you can gather information adequately online? - How difficult is it for you to evaluate the quality of each offer of information online? - Can you report about bad experiences with online services? - How bad do you rank the stigmatization of psoriasis patients online? | Understanding the challenges and dangers |
| Which changes and improvements do you wish for regarding the digital media for psoriasis patients? | - Where do you need help regarding the internet use? - In which online media do you see potential? - Which platforms or social media can you recommend? - What should be paid attention to by actions? | Understanding the chances of online use |
